# Using Real-World Research to Study the Impact of Chronic Daily Therapy Discontinuation in Cystic Fibrosis: The Home-Reported Outcomes in Cystic Fibrosis 2 Study Design

**DOI:** 10.1016/j.chpulm.2024.100080

**Published:** 2024-06-29

**Authors:** Bradley H. Rosen, Kevin J. Psoter, Kathryn A. Sabadosa, Georgene E. Hergenroeder, Lisa L. Bendy, Nell Meosky Luo, Connie Zhang, Clement L. Ren, Cynthia D. Brown

**Affiliations:** a Division of Pulmonary, Critical Care, Sleep, and Occupational Medicine, Department of Medicine, Indiana University School of Medicine, Indianapolis, IN; b Division of Pulmonary, Allergy, and Sleep Medicine (L. L. B.), Department of Pediatrics, Riley Hospital for Children, Indiana University School of Medicine, Indianapolis, IN; c Division of General Pediatrics (K. J. P.), Department of Pediatrics, Johns Hopkins School of Medicine, Baltimore; d Cystic Fibrosis Foundation (K. A. S.), Bethesda, MD; e Division of Pulmonary and Sleep Medicine (G. E. H. and C. L. R.), Department of Pediatrics, Children’s Hospital of Philadelphia, Perelman School of Medicine, University of Pennsylvania, Philadelphia, PA; f Folia Health, Inc. (N. M. L. and C. Z.), Boston, MA

**Keywords:** chronic daily therapies, cystic fibrosis, elexacaftor/ivacaftor/tezacaftor, real-world research, study design

## Abstract

**Background:**

Chronic daily therapies (CDTs) are the foundation of clinical care for people with cystic fibrosis (CF), but these therapies impose considerable burden. In the era of elexacaftor/tezacaftor/ivacaftor (ETI) therapy, it is not clear if CDT discontinuation would lead to a greater decrease in lung function.

**Research Question:**

In people with CF who are taking or about to start ETI, does CDT discontinuation lead to lower lung function at 12 months?

**Study Design and Methods:**

People with CF who are aged 12 years or older and receiving or about to start ETI therapy were included in the Home-Reported Outcomes in Cystic Fibrosis 2 (HERO-2) study, an observational cohort study that used real-world research principles. Recruitment for HERO-2 used a multimodal approach consisting of recruitment sites, referral sites, and community-based strategies. The sole method of study engagement for participants was through the Folia Health application, which participants used to track CDT and symptoms, while completing monthly validated patient-reported assessments. Demographic and clinical data, including spirometry findings, were collected through linkage with the Cystic Fibrosis Foundation Patient Registry (CFFPR). The study was designed to detect a difference of 3% in FEV_1_ % predicted between individuals who did and did not discontinue any CDT.

**Results:**

The multimodal approach to recruitment and broad inclusion criteria allowed HERO-2 to recruit rapidly from > 70 sites, including smaller affiliate centers and community-based outreach sites. The protocol is still being executed, with anticipated results to be published when the complete CFFPR data are available.

**Interpretation:**

To our knowledge, HERO-2 is the first study in the population with CF that was designed using real-world research principles.

**Trial Registry:**

ClinicalTrials.gov; No.: NCT04798014; URL: www.clinicaltrials.gov

**Take-home Points**

**Study Question:** The Home-Reported Outcomes in Cystic Fibrosis 2 study represents, to our knowledge, the first entirely remote, real-world research study in cystic fibrosis as described in this article.

**Results:** Recruitment through a variety of methods including social media, referral, and traditional recruitment allowed rapid enrollment of a large population. Incorporation of patient-reported outcomes and validated questionnaires with outcomes from a patient registry provides real-world evidence with remote data collection.

**Interpretation:** This study design provides the ability to study the use of chronic daily respiratory therapies and their effects on lung function and pulmonary exacerbations in people with cystic fibrosis taking elexacaftor-tezacaftor-ivacaftor.

Chronic daily therapies (CDTs) are a cornerstone of clinical care for people with cystic fibrosis (CF) to preserve lung function and to slow disease progression. However, CDT often presents a significant burden for patients.^1^, ^2^, ^3^ Thus, reducing treatment burden was one of the highest priorities for future research identified in a survey of people with CF and their caregivers.^4^ Given the dramatic improvements in lung function, overall well-being, and nutritional status after initiation of elexacaftor/tezacaftor/ivacaftor (ETI) therapy and its approval for use in > 90% of people with CF in the United States,^5^^,^^6^ an important question now within the CF community is whether the number and type of CDTs can be discontinued safely in individuals receiving ETI therapy.^7^

Recognizing the interest in reducing CDT for people with CF, the Impact of Discontinuing Chronic Therapies in People With Cystic Fibrosis on Highly Effective CFTR Modulator Therapy (SIMPLIFY) trial (ClinicalTrials.gov Identifier: NCT04378153) and CF-Streamlining Treatment or Reducation Medication (CF-STORM) trial (European Union Drug Regulating Authorities Clinical Trials Database Identifier: 2020-005864-77) randomized controlled trials (RCTs) were designed to test whether discontinuation of the commonly prescribed mucolytics dornase alfa (DNase) or hypertonic saline (HTS) in people with CF receiving ETI therapy would result in greater lung function decline compared with continuation of the therapy. Completed in 2022, results of the SIMPLIFY trial demonstrated that in people with CF with preserved lung function, no clinically significant differences were found in lung function at 6 weeks between individuals randomized to either continuation or discontinuation for either therapy (DNase or HTS), providing evidence that either treatment may be discontinued safely in the shortterm.^8^ The ongoing CF-STORM study was designed to evaluate the longer-term effects of discontinuation of DNase, HTS, or both on lung function over a 52-week period in a population of people with CF that includes a wider range of disease severity than the SIMPLIFY trial. Taken together, the SIMPLIFY and CF-STORM trials will provide the first high-level evidence regarding the effects of DNase and HTS discontinuation, which subsequently may be used to inform clinical care guidelines. Although characterized by differing study populations, data collection protocols, and durations of observation, the randomization of discontinuation of CDT is the important unifying aspect for these studies.

The protocolized nature of RCTs designed to test discontinuation of CDT can be challenging to conduct. People with CF may not want to participate in an RCT or to discontinue a CDT, may have preferences for which CDT to discontinue, or may decide to discontinue CDT based on shared decision-making with clinicians. For these reasons, individuals may prefer to participate in studies that are less burdensome and in which changes to CDT are not randomized. Real-world research studies provide an opportunity to fill this participation gap while evaluating real-time treatment discontinuation in individuals^9^, ^10^, ^11^ in a manner that may reflect real-life clinical practice and patient behaviors more closely.^10^^,^^12^^,^^13^ A primary component of many real-world research studies is the use of real-world data, including electronic health records, clinical data collected as part of routine care, and patient registries, as well as patient-reported outcomes (PROs). Although RCTs recruit highly selected patients in standardized settings to ensure internal validity, real-world research provides a complementary approach by emphasizing generalizability in real-world settings with real-life clinical management.^9^^,^^14^ The CF population is a model population in which real-world research can be conducted because of the availability of the Cystic Fibrosis Foundation Patient Registry (CFFPR) and a robust clinical care network. The Home-Reported Outcomes in Cystic Fibrosis 2 (HERO-2) study (ClinicalTrials.gov Identifier: NCT04798014) was funded by the Cystic Fibrosis Foundation (CFF) and initiated to understand CDT use further in people with CF receiving ETI therapy. To the best of our knowledge, it is ta first-of-its-kind real-world research study in people with CF that incorporates fundamental real-world research design principles, including broad inclusivity, minimal clinical study visits, data collection through the CFFPR, and use of PROs captured remotely (Fig 1). The purpose of this article is to describe the design aspects of the HERO-2 study.

**Figure 1:**
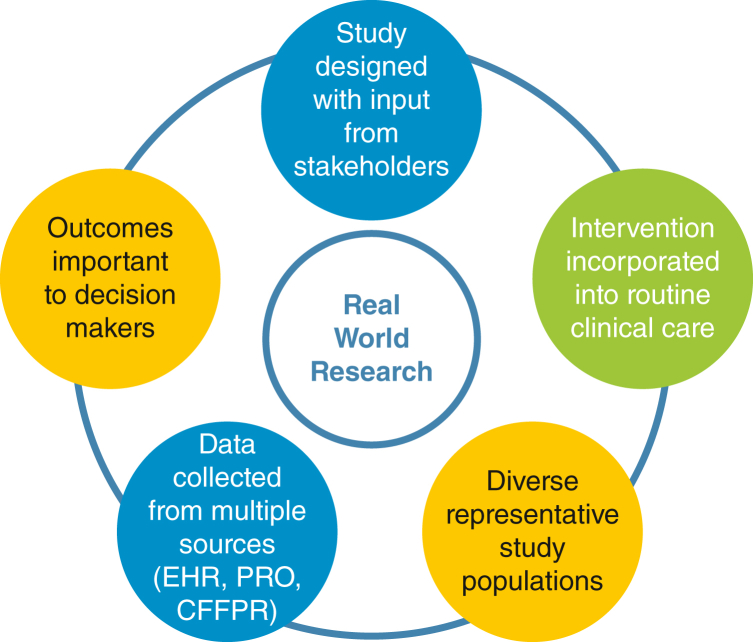
Diagram showing principles of real-world research. Real-world research prioritizes the use of data collected in the routine care of the participants (EHR or registry data) and PROs, rather than at standardized study visits, and has broad inclusion criteria to capture diverse populations in the study. It is common to have study design input from stakeholders and relevant advocacy groups in some real-world research. CFFPR = Cystic Fibrosis Foundation Patient Registry; EHR = electronic health record; PRO = patient-reported outcome.

## Study Design and Methods

### Study Aims

The aims of the HERO-2 are: (1) to determine the proportion and clinical characteristics of people with CF receiving ETI therapy who make changes to the CDT after initiation of ETI and (2) to evaluate the effect of these changes in CDT on lung function, IV antibiotic-treated pulmonary exacerbations (PExs), and symptoms during a 12-month follow-up period.

### Study Design and Participants

The HERO-2 trial is a 12-month observational cohort study that includes remote data collection procedures entered by participants with linked patient-level data collected from the CFFPR. Individuals were eligible to participate in the HERO-2 trial if they were aged 12 years or older at the time of enrollment, had a diagnosis of CF at a CF care center, were about to start or were receiving ETI therapy for a US Food and Drug Administration-labelled indication, had access to a phone or computer with an internet connection to engage in study procedures, and previously consented to have their clinical data entered in the CFFPR (Table 1). Exclusion criteria included lack of access to necessary technology to participate in data entry, prior solid organ transplantation, limited English proficiency that could interfere with study participation, or a combination thereof. Because the HERO-2 trial enrollment coincided with the SIMPLIFY study, SIMPLIFY participants could participate in HERO-2 only after completion of the randomized 6-week phase of that RCT.

**Table 1:** HERO-2 Trial Inclusion and Exclusion Criteria

Inclusion Criteria	Exclusion Criteria
• ≥ 12 y of age at the time of enrollment	• No access to the necessary technology
• Diagnosis of CF made at a CF care center	• Recipient of a solid organ transplantation
• About to start, or currently taking, ETI therapy	• Limited English proficiency
• Smartphone or computer with internet access	
• Willing to enter data daily for 12 mo	
• Consent to have data entered into the Cystic Fibrosis Foundation Patient Registry	

CF = cystic fibrosis; ETI = elexacaftor/tezacaftor/ivacaftor; HERO-2 = Home-Reported Outcomes in Cystic Fibrosis 2.

### Folia Health Application

The sole method of study engagement for participants is through the Folia Health application, a cloud-based system that can be accessed by mobile device application or web browser and is intended for use for personal health tracking, research participation, or both. This platform allows centralized informed consent to be completed by the user without the need for direct research staff involvement.

### Recruitment

A multimodal recruitment strategy included three distinct approaches to enroll a broader population of people with CF, including those who typically may not participate in CF studies: (1) CF care centers with dedicated research coordinators (RCs); (2) referral from CF clinics; and (3) community-based announcements through social media.

#### Recruitment Sites

Twenty-three recruitment sites used RCs who approached potential participants during routine clinical encounters or through phone calls or e-mail. RCs described the study and provided an enrollment code for the individual to access the HERO-2 study enrollment portal in the Folia Health application. In addition, RCs contacted participants at 1, 4, 7, and 10 months to answer any study-related questions and to provide encouragement to remain in the study. No study data were collected during these calls.

#### Referral Sites

Fifty-seven referral sites were asked to promote the study in their clinics, which included posting referral flyers, e-mailing study information to potential participants, having team members provide study information to potential participants during a clinic visit, or a combination thereof. Referral sites did not contact participants after enrollment and did not know which individuals at their site chose to participate.

#### Community-Based Recruitment

Community-based recruitment included messages to existing Folia Health users and announcements through institutional review board-approved materials posted on CFF-hosted social media channels (Facebook, Instagram, and X) and other forums such as the CFF Clinical Trials Finder website, Together newsletters, and Community Voice publications. The Together newsletter is distributed to all those who request to receive CFF national emails, while Community Voice is a monthly online newsletter managed by and for people with CF.

### Study Procedures

#### Enrollment, Remote Consent, and Data Collection

All participants independently enrolled and completed the centralized informed consent forms through the Folia Health application. After giving informed consent, participants completed the study initiation survey (called the baseline survey). This included the self-reported current (baseline) treatment plan, ETI start date, prior CDT changes after ETI initiation, and selection of which symptoms and measurements to track. Cough characteristics, including severity (0-10 scale) and features (mucus production and quality), were required as part of the routine tracking questions. Users could choose additional symptom categories for routine tracking or add on a per-day-basis as their condition changed. The Folia Health application suggests additional CF-related items to track as the first tier of options, but also allows the participant to write in their own items. Symptom categories included: pulmonary, gastrointestinal, mental health, and others. As part of the HERO-2 trial, participants were required to track a minimum of 3 d/wk (12 d/mo) to be eligible for and to receive honoraria. Tracking could be carried out for a single day at a time or for multiple days at a time through Folia’s batch tracking feature. The batch tracking mechanism allowed participants to report identical data points (no changes) for up to 1 week in a single entry vs entering the same data day after day. Use of this method alone was not sufficient to meet criteria for honoraria.

At the end of each calendar month, participants completed a monthly review survey in which they reported any changes to the treatment regimen and occurrence of any PExs in the previous month. This monthly survey also included two validated patient-reported assessments: the Patient-Reported Outcome Measurement Information System, a 10-item measure of health status that spans physical, mental, and social domains,^15^ and the Cystic Fibrosis Respiratory Symptom Diary-Chronic Respiratory Infection Symptom Score, a 16-item CF-specific questionnaire that covers respiratory symptoms alongside emotional and other aspects of health.^16^

At the end of the 12-month study, participants completed an end-of-study survey, which included a participant satisfaction survey, as well as the opportunity to provide feedback from about study experiences. Individuals also provided information about social determinants of health at this time, specifically concerning the dimensions of stable housing, transportation, and food security.

#### Cystic Fibrosis Foundation Patient Registry

**Figure 2:**
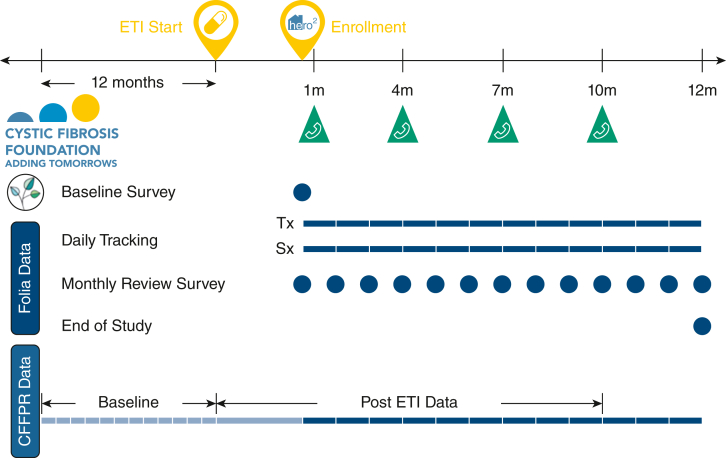
Design schematic of the Home-Reported Outcomes in Cystic Fibrosis 2 (HERO-2) study. Folia data acquisition overlaps and integrates with CFFPR data to allow for a comprehensive assessment of patient-level chronic daily therapies and clinical outcomes. PPR = Cystic Fibrosis Foundation Patient Registry; ETI = elexacaftor/tezacaftor/ivacaftor; Tx = treatments; Sx=symptoms

At study entry, participants provided their name and date of birth in the Folia Health application, which then was used to link to clinical data in the CFFPR. The CFFPR includes detailed demographics (age, sex, race, ethnicity), clinical information (spirometry findings, anthropometric results, CFTR mutations, respiratory microbiology findings, complications, and IV antibiotic-treated PExs), and treatments prescribed by clinicians collected as part of routine clinical encounters and care episodes.^17^ Although participants reported ETI start date in the baseline survey, the CFFPR also collects the date of ETI prescription and start date. Figure 2 summarizes the timing of data collection during participation in the study. Only individuals enrolled at recruiting sites received the phone calls designated at 1, 4, 7 and 10 months of follow-up.

#### Data Management and Security

The Folia Health platform is designed to comply with US Code of Federal Regulations 21 Parts 11, 50, and 56, with electronic consent capabilities. The collection and storage of de-identified participant responses uses a participant number identifier stored in Folia's secure Amazon Web Services framework, in which Folia encrypts data during transfer and when stored.

### Outcomes

The primary outcome for the HERO-2 trial is the change in FEV_1_ % predicted from baseline to the 1-year follow-up in those who discontinued a CDT compared with those who did not discontinue a CDT. This change in FEV_1_ % predicted is obtained from the CFFPR by matching the Folia baseline survey dates to the nearest preceding spirometry data (baseline FEV_1_ % predicted) and comparing it with the spirometry findings closest to 12 months after the initial value. Because CFFPR data capture and reporting depend on the cadence of clinical care delivery independent of the study, the timing does not necessarily align with study enrollment. Discontinuation of any CDT (high-frequency chest wall oscillation, chest physiotherapy, oscillating positive expiratory pressure, exercise, mucolytics, inhaled antibiotics, and azithromycin) is the primary exposure variable and included discontinuation after ETI initiation as reported in the study initiation baseline survey and at the monthly review survey follow-up intervals. Tracking data were not included in the primary exposure because reliability and completeness could not be determined a priori. Table 2 describes all outcomes (primary and secondary) that will be collected.

**Table 2:** Overview of HERO-2 Study Outcomes

**Primary outcome**
Absolute change in FEV_1_ % predicted from ETI therapy initiation compared with 12-mo follow-up
**Secondary outcomes**
Proportion of participants reporting changes to at least one chronic daily respiratory medication or airway clearance treatment during the 12-mo follow-up period
Any occurrence and rate of IV antibiotic-treated CF PEx during follow-up (CFFPR outcome)
Time to first IV antibiotic-treated PEx after discontinuation of CDTs
Health-related quality of life (Patient-Reported Outcome Measurement Information System)
CF-specific symptoms score (Cystic Fibrosis Respiratory Symptom Diary-Chronic Respiratory Infection Symptom Score)
Cough score

CDT = chronic daily therapy; CF = cystic fibrosis; CFFPR = Cystic Fibrosis Foundation Patient Registry; ETI = elexacaftor/tezacaftor/ivacaftor; HERO-2 = Home-Reported Outcomes in Cystic Fibrosis 2; PEx = pulmonary exacerbation.

### Statistical Analysis

The primary statistical procedures will compare change in FEV_1_ % predicted from baseline to 1 year between participants who discontinued any CDT to those who did not discontinue a CDT using multivariable linear regression. Generalized linear models with appropriate distributions and link functions then will be used to compare PEx outcomes (occurrence, rate, and time to first PEx) between participants who did and did not discontinue CDT. Generalized estimating equation-based linear regression will be used to evaluate the association of treatment discontinuation with change in Cystic Fibrosis Respiratory Symptom Diary-Chronic Respiratory Infection Symptom Score scores, Patient-Reported Outcome Measurement Information System scores, and cough score during follow-up. All models will be adjusted for individual-level demographic, clinical, and disease characteristics. Inverse probability weighting will be used to account for the nonrandomized nature of CDT discontinuation. Descriptive statistics will be used to summarize CDT and concordance of self-reported CDT use to CDTs prescribed from the CFFPR.

### Sample Size

Sample size is based on detecting a clinically significant difference of 3% in the change in FEV_1_ % predicted from baseline to the 12-month follow-up between the estimated 30% of individuals who make a change to the CDT and the 70% whose treatment plan remained unchanged using a multivariable linear regression model. A difference of 3% in FEV_1_ % predicted was chosen because it was sufficient potentially to change clinical management and was closely aligned with the SIMPLIFY study.^8^ The assumption that 30% of participants would report a change in CDT was based on survey data collected as part of the planning phase for the HERO-2 study. A total of 860 participants were required to test the null hypothesis of no difference between FEV_1_ % predicted at an α value of .05 and power of 0.80. The sample size was derived assuming an anticipated 30% dropout or nonparticipation rate and a squared multiple correlation coefficient of 0.3 when covariates are included in the final multivariable model.

### Study Status

**Figure 3:**
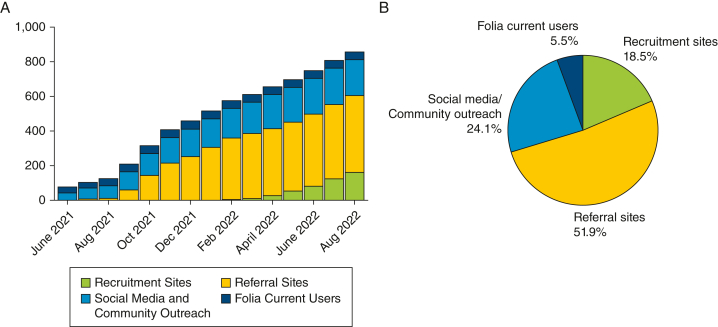
A, B, Bar graph showing Home-Reported Outcomes in Cystic Fibrosis 2 enrollment from June 2021 through August 2022 (A) and circle graph showing breakdown by method of recruitment and sources (B). Social media and outreach included Cystic Fibrosis Foundation channels, cystic fibrosis-related community organizations, and conferences.

The study protocol was approved by the Advarra Institutional Review Board on May 7, 2021 (Identifier: Pro00048780). Community-based enrollment began in June 2021, followed by referral sites in August 2021, and recruitment sites in March 2022. The study achieved its recruitment goal in August 2022 (Fig 3). The primary outcomes will be available after the release of the 2023 CFFPR data, which is anticipated in 2024.

## Discussion

The HERO-2 trial was designed to understand patterns of CDT discontinuation and its effects in people with CF after initiation of ETI therapy. The timely nature of this study will help to inform and support the shared decision-making process of CDT discontinuation and will complement findings from completed and ongoing RCTs, as well as other observational studies evaluating CDT discontinuation. In addition, the longitudinal collection of PROs, including symptoms and quality of life, will fill current gaps in longer-term experiences of people with CF after ETI therapy initiation, as well as CDT discontinuation. The SIMPLIFY trial has demonstrated that discontinuation of either hypertonic saline or DNase was noninferior to continuing treatment in people with CF who are clinically stable on CFTR modulators. It is particularly notable that participants in the SIMPLIFY trial had a mean lung function of approximately 97% predicted and participants were highly adherent to assigned therapies, including 99% adherence to ETI therapy and > 95% adherence to the assigned discontinuation group.^8^ Generalizability of these findings to the greater CF community may be limited, in particular for individuals less adherent to ETI therapy or those with lower lung function. By leveraging the unique aspects within the CF community, the HERO-2 trial will build on these results and will evaluate CDT discontinuation in a real-world setting.

To the best of our knowledge, the HERO-2 trial is the first observational study to incorporate real-world research principles into the design and represents a major commitment to the conduct of real-world research in the population with CF. The HERO-2 trial was designed in a collaborative manner with a particular emphasis on integrating aspects within the design to encourage widespread participation, minimizing study burden, and collecting clinical data to allow for comparison across studies. The four innovative design aspects that the HERO-2 trial adopted to achieve these goals included: (1) broad inclusion criteria of patients within the United States, (2) a multimethod recruitment strategy, (3) remote collection of CDTs and PROs, and (4) linkage of study participants with clinical data from the CFFPR.

Inclusion criteria for the HERO-2 trial was purposefully not restrictive to encourage recruitment and participation of a diverse study population with the goal of increasing generalizability of results of CDT discontinuation in a real-world setting. Participants completed all study procedures through the Folia Health application, with no requirement for clinical research visits. This decentralized and less burdensome study design may contribute further to inclusion of a more diverse population in other studies. Second, the approach to recruitment across CF care centers (both recruitment and referral centers) and through the CF community resulted in achieving the required sample size in a timely fashion (Fig 3). Subsequent comparison of characteristics of individuals by recruitment method are planned and may provide guidance for future study design, particularly when combined with an understanding of participant engagement within each group. The HERO-2 trial demonstrates that these methods of recruitment, enrollment, and consent are feasible to conduct in people with CF and may provide a more cost-effective approach that future studies could adopt, even when large sample sizes are required. Study engagement during the 1-year follow-up will be detailed and results of the end of study survey will be reported, both of which may help to inform the design of similar studies. Finally, the use of the CFFPR allowed the HERO-2 trial to be conducted remotely without the need for in-person encounters for demographic and clinical outcomes.

The study has several limitations. First, this study is limited to individuals aged 12 years and older who are eligible and have initiated ETI therapy, who had internet access, and who could interact with the application in English. Therefore, results will be generalizable only to this population and do not include individuals receiving other modulator therapies, those not eligible for ETI therapy, or those who are unable to access the Folia Health application. Further, the representativeness of the HERO-2 study population, compared with the larger population of individuals eligible for ETI therapy, will be explored and may limit generalizability further. Second, the HERO-2 trial relies on clinical data that are recorded in the CFFPR. It is possible that baseline or follow-up spirometry findings and other clinical data are unavailable because of missed or telehealth clinical encounters. Similarly, it is possible that some HERO-2 participants will not be linked successfully to the CFFPR or did not meet eligibility criteria (eg, prior solid organ transplantation). Finally, the primary exposure of interest was self-reported discontinuation of CDT, which may introduce misclassification if CDTs are not reported accurately.

In summary, the HERO-2 trial is a novel study in the CF population that incorporated real-world research principles into its design with the goal of better understanding CDT in people with CF and outcomes after discontinuation of CDT in those eligible for ETI therapy. The multimethod recruitment strategy and remotely captured patient-reported data complemented by data collected as part of the CFFPR are unique aspects that may be considered and incorporated into future studies in the population with CF.

## Funding/Support

This study was funded by a grant from the Cystic Fibrosis Foundation (BROWN21AB0)

## Financial/Nonfinancial Disclosures

The authors have reported to *CHEST Pulmonology* the following: K. A. S. is employed by the CFF, a nonprofit entity. N. M. L. and C. Z. are employees at Folia Health, which is a for-profit entity. C. D. B reports a relationship with Vertex Pharmaceuticals Incorporated that includes: funding grants. C. D. B reports a relationship with the Cystic Fibrosis Foundation that includes: funding grants. C. L. R. reports a relationship with Vertex Pharmaceuticals Incorporated that includes: funding grants. C. L. R. reports a relationship with the Cystic Fibrosis Foundation that includes: funding grants. None declared (B. H. R., K. J. P., G. E. H., L. L. B.,).
